# Superactive human leptin antagonist (SHLA), triple Lan1 and quadruple Lan2 leptin mutein as a promising treatment for human folliculoma

**DOI:** 10.1007/s00280-017-3423-5

**Published:** 2017-08-31

**Authors:** E. Fiedor, E. L. Gregoraszczuk

**Affiliations:** Department of Physiology and Toxicology of Reproduction, Institute of Zoology and Biomedical Research, Jagiellonian University, Gronostajowa 9, 30-387 Kraków, Poland

**Keywords:** Folliculoma cells, Leptin receptor antagonists, ObR expression, ERα/ERβ expression, Cell cycle protein expression

## Abstract

**Purpose:**

There are no data showing a direct correlation between obesity and increased blood leptin levels with folliculoma. Moreover, folliculoma is not the best studied among other ovarian cancer types. We investigated whether oestradiol can modulate ObR expression in some oestrogen-responsive tissues and that leptin exerts its activity not only via the leptin receptor but also through cross talk with other signalling systems. We hypothesise that blocking ObR expression could be a novel treatment for gonadal ovarian cancer.

**Methods:**

We evaluated the effect of SHLA, Lan1 and Lan2 blockers on cell proliferation (BrdU incorporation assay), ObR and ERα/β gene expression (qPCR), oestradiol secretion (ELISA) and cell cycle protein expression (Western blot) in the non-cancerous cell line HGrC1 and two granulosa cancer cell lines: the juvenile form (COV434) and the adult form (KGN).

**Results:**

ObR gene expression in cancer cell lines was 50% higher than in the non-cancer cells. Lan-1 and Lan-2 decreased ObR expression in COV434, while it had no effect in KGN cells. Higher ERβ expression in non-cancer and higher ERα expression in both cancer cell lines was noted. SHLA and Lan-1 changed the ratio towards greater expression of ERβ, characteristic of non-cancer granulosa cells. All ObR antagonists in HCrC1 and KGN but only Lan-2 in COV434 reversed leptin-stimulated proliferation. In both non-cancer and cancer granulosa cells, leptin acts as a cyclinD/cdk4, cyclin A/cdk2 and E2F inhibitor.

**Conclusion:**

These results indicate that SHLA and Lan2 are promising leptin receptor inhibitors that can eliminate the negative effects of leptin. These compounds should be considered in further ex vivo studies on the cancer microenvironment.

**Graphical abstract:**

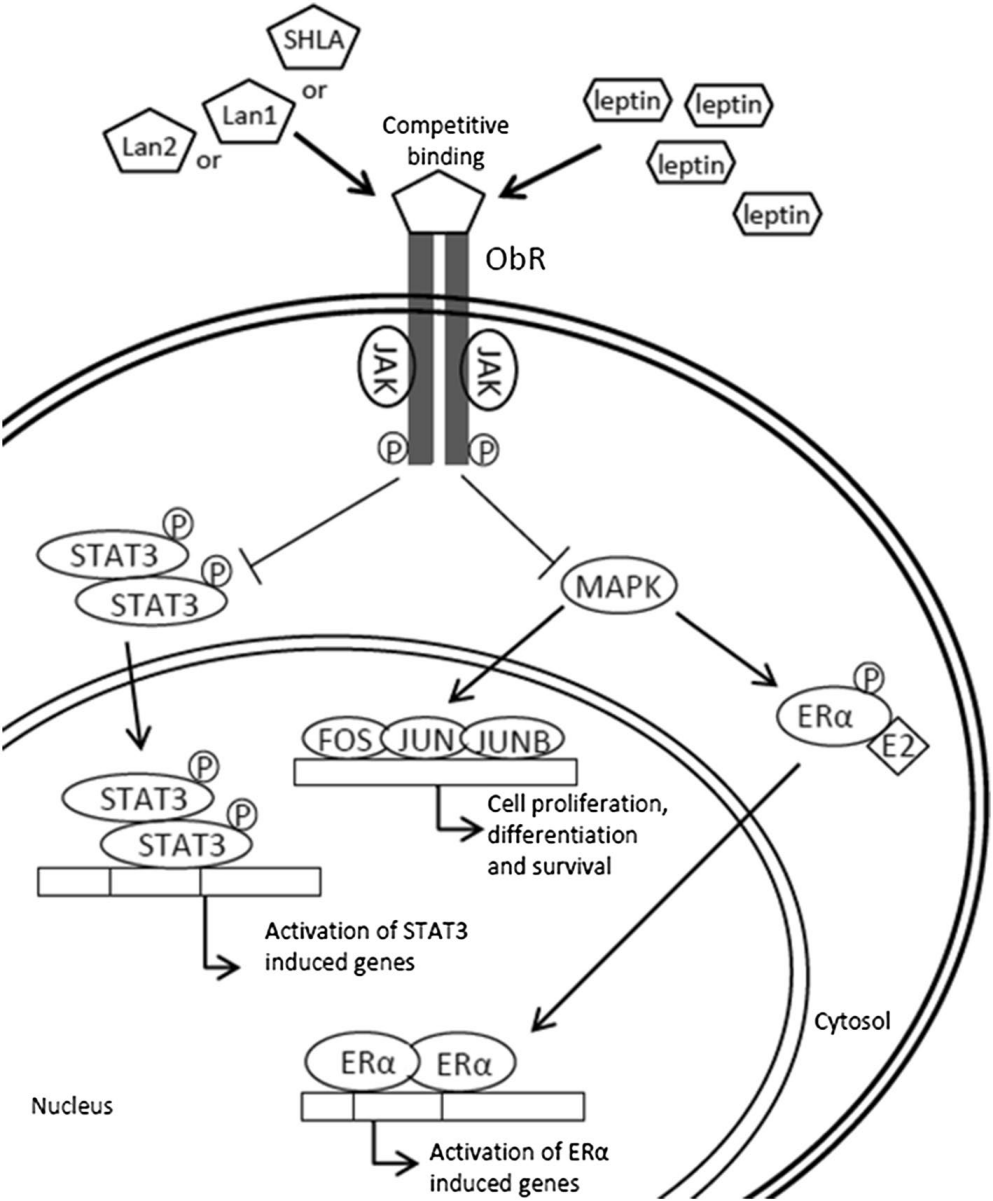

## Introduction

Leptin is a small (16 kDa) protein produced and secreted by adipose tissue, which is involved in appetite regulation, bone formation and reproductive function. Recent studies have demonstrated that this hormone stimulates growth, migration, invasion and angiogenesis in tumour cell models, suggesting that leptin is capable of promoting an aggressive cancer phenotype [1]. Epidemiological studies have indicated a positive correlation between obesity and an increased risk of several types of cancer [2]. Serum leptin levels have been reported to be higher in overweight and obese women than in women with normal weight. In obese individuals, leptin levels can reach 40 ng/mL, which is up to ten times higher than in normal weight people [3, 4]. Cancer risk is higher among overweight and obese people, with an increased risk of 16 and 30%, respectively [5]. Moreover, leptin and its receptors are over-expressed in different human cancers [1]. Leptin has been proposed as one of the six markers of ovarian cancer [6]. Uddin et al. revealed a significant association between ObR overexpression and poor survival rates in 59.2% of epithelial ovarian cancer cases [7].

Granulosa cell tumours constitute the second largest group of ovarian tumours (approximately, 25–30%), with tumours derived from epithelial cells accounting for approximately 70% [8]. Granulosa cell tumours can be divided into two histopathological forms: a mature form, diagnosed most frequently in peri-menopausal women (95%), and a juvenile form, diagnosed in young women and girls who have aged prematurely (5% of cases) [9]. Despite the fact that granulosa cell tumours can be successfully treated by surgery, relapses are often observed and further adjuvant treatment is still not possible.

To our knowledge, there are no data showing a direct correlation between obesity and folliculomas. Moreover, folliculoma is not well studied among the ovarian cancer types, although both the short (ObRa) and long (ObRb) forms of the leptin receptor are present in human granulosa cells [10, 11]. Löffler et al. [10] showed that, in polycystic ovaries, leptin-positive cells were noted both in the hypertrophied theca layer and in the luteinised granulosa layer. Taking into consideration that leptin at a supraphysiological concentration, as noted in obese women, has stimulatory effects on testosterone secretion characteristic of polycystic ovarian syndrome correlated with obesity [12], we hypothesised that, as in the case of epithelial ovarian cancer, ObR overexpression in granulosa cell tumours could be correlated with the incidence of granulosa cell cancer, and leptin receptor blockers might be used as an adjuvant therapy.

Currently, several groups of scientists are working on the synthesis of molecules that block ObR. A number of leptin receptor antagonists have been synthesised for therapeutic use, with several completing pre-clinical testing [13], indicating their possible use in anticancer therapy. In previous studies using different epithelial cancer cell lines, we showed that SHLA and quadruple leptin mutein, Lan2 (L39A/D40A/F41A/I42A), had no effect on non-cancerous HOSEpiC cell proliferation [14]. However, both antagonists reversed the stimulatory effect of leptin on metastatic carcinoma CaOV-3 cell proliferation to control levels and even below control levels in chemoresistant OVCAR-3 cells. Leptin receptor antagonists have been investigated in breast and prostate cancers, which mainly are hormone dependent. It has been shown that Aca1, Allo-aca and D-ser can inhibit leptin-stimulated proliferation in MCF-7 breast cancer cells [15]. Another leptin receptor antagonist, LDFI (Leu-Asp-Phe-Ile), is also able to inhibit the proliferation of MCF-7 cells in vitro and in vivo [16]. The antagonist Lan1 is able to inhibit the phosphorylation of leptin-signalling proteins Jak2, ERK1/2 and Akt, in PC3 and DU145 prostate cancer cell lines [17]. In a previously published study using epithelial ovarian cancer cell lines, we investigated the effect of SHLA and Lan2 on the JAK/Stat3, MAPK/ERK and PI3K/Akt pathways and showed an inhibitory effect of SHLA on all tested signalling proteins in OVCAR-3 cells and of Lan2 on Stat3 and ERK1/2 proteins in CaOV-3 cells [14]. These data point to a similar signalling pathway in the antagonistic effects of leptin receptor blockers.

In the present study, we evaluated the effect of leptin and three of its receptor antagonists: Lan1 (L39A/D40A/F41A mutant), Lan2 (L39A/D40A/F41A/I42A mutant) and SHLA (D23L/L39A/D40A/F41A mutant) on leptin and oestradiol receptor gene and protein expression, cell proliferation including cell cycle protein expression, caspase-3 activity and oestradiol secretion in two granulosa tumour cell lines. The two cell lines were COV434, representing the juvenile form of granulosa tumour, and the steroidogenic human ovarian granulosa-like tumour (KGN), representing the adult type of this cancer (corresponding to peri- to post-menopausal age). The human immortalised non-luteinised granulosa cell line HGrC1 was used as the control.

Based on the fact that leptin exerts its activity not only through the leptin receptor (ObR), but also through cross talk with other signalling systems implicated in tumour genesis [18, 19], in this study we focused our attention on the relationship between the leptin/ObR axis and oestrogen receptors (ERα/β).

Taking into consideration that oestradiol can modulate ObR expression in some oestrogen-responsive tissues, we hypothesised that blocking ObR expression could be a novel treatment for granulosa ovarian cancer.

## Materials and methods

### Reagents

Dulbecco’s modified Eagle’s medium/Nutrient Mixture F-12 (DMEM/F-12) was obtained from Gibco by Thermo Fisher Scientific (Waltham, MA, USA). DMEM, foetal bovine serum (FBS, heat inactivated), penicillin and streptomycin were obtained from Sigma Chemical Co. (St. Louis, MO, USA). All applied media were oestradiol free. Leptin was obtained from Sigma Chemical Co. (St. Louis, MO, USA). Leptin receptor antagonists (SHLA, Lan1 and Lan2) were obtained from Protein Laboratories Rehovot (PLR) Ltd. (Rehovot, Israel). All antagonists have the same specificity. They interact with the cytokine homology domain 2 in the leptin receptor and do not interact with IGD (immunoglobulin-like domain) of the receptor, as the 39–40, 39–41 or 39–42 Ala mutation abolished this interaction [20].

### Cell culture

HGrC1 (human non-luteinised granulosa cell line) cells were a gift from Dr Ikara Iwase (Nagoya University, Japan) and cultured according to the protocol described by Bayasula et al. [21]. HGrC1 may possess the characteristics of granulosa cells in early stage follicles. A human immortalised non-luteinised granulosa cell line (HGrC1) originally derived from mural granulosa cells expresses the FSH receptor and is responsive to the transforming growth factor (TGF)-β superfamily and FSH, retaining its original granulosa cell character and function. HGrC1 might also be capable of growth transition from a gonadotrophin-independent status to gonadotrophin-dependent one, but they are not capable of undergoing luteinisation.

COV434 cells were obtained from the Sigma Chemical Co. (St. Louis, MO, USA). The biological characteristics of this cell line include production of 17β-oestradiol in response to FSH, absence of the LH receptor, no luteinisation capability and the presence of specific molecular markers of apoptosis enabling the induction of follicular atresia [22].

KGN cells were obtained from Masatoshi Nomura and Hajime Nawata, Kyushu University, Japan. With luteinisation capability, they constitute a useful model for understanding the regulation of steroidogenesis, cell growth and apoptosis in human granulosa cells [23].

HGrC1 and COV434 cells were routinely cultured in DMEM + 2 mM glutamine + 10% FBS. KGN cells were routinely cultured in DMEM/F-12 + 10% FBS. Cells were grown in 75 cm^2^ tissue culture dishes (Nunc, Denmark) in a 37 °C incubator with a humidified mixture of 5% CO_2_:95% air.

### Experimental procedure

#### qPCR analysis

Basal ObR gene expression and expression of the ObR gene under the influence of leptin and leptin antagonists was determined by qPCR. Cells were seeded into 96-well culture plates at a density of 5 × 10^3^ cells/well (HGrC1), 8 × 10^3^ cells/well (COV434) and 1.5 × 10^4^ cells/well (KGN) taking into consideration the size of the cells and the population doubling time. The next day, the medium was changed and cells were treated with leptin at a dose of 40 ng/mL and SHLA, Lan1 and Lan2 at a dose of 1000 ng/mL with leptin at a dose of 40 ng/mL for 24 h. Doses of leptin were chosen based on literature data [3, 4]. Total RNA isolation and cDNA synthesis was performed using the TaqMan Gene Expression Cell-to-CT Kit (Applied Biosystems, Carlsbad, CA, USA) in accordance with the manufacturer’s protocol. Amplifications were performed using the StepOnePlus system (Applied Biosystems, Carlsbad, CA, USA) and the TaqMan Leptin Receptor primer (Cat. No. Hs00174497_m1), oestrogen receptor α primer (Hs00174860) and oestrogen receptor β primer (Hs01100353) in combination with the TaqMan Gene Expression Master Mix (Applied Biosystems, Carlsbad, CA, USA), in accordance with the manufacturer’s instructions.

A PCR was performed using a final volume of 20 μL, including 100 ng/reaction cDNA. PCR conditions were as follows: pre-incubation (2 min at 50 °C and 10 min at 95 °C), amplification for 40 cycles (15 s at 95 °C and 1 min at 60 °C). The relative expression of genes was normalised against the endogenous reference gene GAPDH (Human GAPD Endogenous Control, number 4333764F) (ΔCq) and converted to relative expression using the 2^−ΔΔCq^ method. The results are expressed as relative values (RQ).

#### Western blot analysis

Cells were plated into 24-well plates at a density of 2.5 × 10^4^ (HGrC1 cells), 3 × 10^4^ (COV434 cells) and 6 × 10^4^ (KGN cells) and allowed to attach overnight. The following day, the media were changed and cells were treated with 40 μg/mL leptin alone or in combination with 1000 μg/mL SHLA, Lan1 or Lan2. To examine cell cycle protein expression, cells were incubated for 48 h. After incubation, cells were washed with ice-cold PBS and lysed with Laemmli lysis buffer (Sigma Chemical Co., St. Louis, MO, USA). The lysed cells were then scraped, transferred to microtubes and stored at −70 °C until analysis.

Prior to analysis, samples were sonicated and centrifuged at 15,000×*g* for 15 min at 4 °C. The quantity of protein was determined using the Bradford method and the clear supernatant was used for electrophoresis. Equal amounts of protein (100 µg) from each treatment group were separated by SDS-PAGE and transferred to PVDF membranes using a Bio-Rad Mini-Protean 3 apparatus (Bio-Rad Laboratories Inc., Hercules, CA, USA). The blots were blocked for 1 h in 5% BSA with 0.1% Tween-20 in 0.02 M TBS buffer. Blots were incubated overnight with primary antibodies specific to ObR (ab5593, Abcam, Cambridge, Great Britain) at a dilution of 1:2000, cyclin D1 (#2978, Cell Signaling Technology Inc., Beverly, MA, USA), cdk4 (#12790), cdk2 (#2546), cyclin A2 (#4656) at a 1:1000 dilution and E2F-1 (sc-251 Santa Cruz Biotechnology Inc., Santa Cruz, CA, USA), E2F-2 (sc-633), ERα (sc-8002) and ERβ (sc-6822) at a dilution of 1:200. After incubation with the primary antibody, the membranes were washed three times with 0.1% Tween-20 in 0.02 M TBS buffer and incubated for 1 h with an appropriate horseradish peroxidase-conjugated secondary antibody (#7074 or #7076, Cell Signaling Technology Inc., Beverly, MA, USA; dilution 1:2000).

β-Actin was used as an internal loading control; membranes were washed for 30 min in stripping buffer (0.25 M glycine, 1% SDS, pH 2) and reprobed by overnight incubation with primary antibodies specific to β-actin (A5316, Sigma Chemical Co., St. Louis, MO, USA; dilution 1:2000) and for 1 h with a horseradish peroxidase-conjugated secondary antibody (P0447 DAKO, Glostrup, Denmark; dilution 1:5000).

Immunopositive bands were visualised using Western Blotting Luminol Reagent (Santa Cruz Biotechnology Inc., Santa Cruz, CA, USA) and ChemiDoc™ XRS+ System (Bio-Rad Laboratories Inc., Hercules, CA, USA). Relative levels of protein expression were determined using ImageJ software (US National Institutes of Health, Bethesda, MD, USA). Individual protein levels were normalised to β-actin controls and the ratio of protein to β-actin was normalised to 1 in the untreated control group.

#### Cell proliferation BrdU assay

DNA synthesis in proliferating cells was determined by measuring bromodeoxyuridine (BrdU) incorporation with the commercial Cell Proliferation ELISA System (Roche Molecular Biochemicals, Mannheim, Germany). The cells were seeded in 96-well culture plates at a density of 5 × 10^3^ cells/well (HGrC1), 8 × 10^3^ cells/well (COV434) and 1.2 × 10^4^ cells/well (KGN). Leptin was added at a concentration of 40 ng/mL. Leptin receptor antagonists were added at concentrations of 10, 100 or 1000 ng/mL, with leptin at a concentration of 40 ng/mL. Cells were cultured for 48 h with repeated exposure; the culture medium was changed daily and fresh compounds added. After 48 h, the medium was removed and cells were incubated for 3 h with a BrdU labelling solution, containing 10 µM BrdU. The assay was performed according to the manufacturer’s instructions. Absorbance values were measured at 450 nm using an ELISA reader (ELx808 BIO-TEK Instruments, Vinooski, VT, USA). The culture medium alone was used as a control for non-specific binding.

#### Caspase-3 activity assay

HGrC1 cells were seeded in 96-well culture plates at a density of 5 × 10^3^ cells/well, COV434 cells at a density of 8 × 10^3^ cells/well and KGN cells at a density of 1.2 × 10^4^ cells per well. Leptin receptor antagonists were added at concentrations of 10, 100 or 1000 ng/mL, with leptin at a concentration of 40 ng/mL. Cells were cultured for 48 h with repeated exposure. The culture medium was changed daily and fresh compounds were added. After exposure, the medium was removed and the plates were stored at −70 °C. Cells were lysed in buffer containing 50 mM HEPES, 100 mM NaCl, 0.1% CHAPS, 1 mM EDTA, 10% glycerol and 10 mM DTT. The assay was carried out by adding 20 M of Ac DEVD-AMC, a substrate for the fluorometric determination of caspase-3 activity. Reaction mixtures were incubated at 37 °C. After 3 h, fluorescence was measured at 360 nm excitation and 460 nm emission using a micro-ELISA plate reader (Bio-Tek Instruments).

#### Oestradiol secretion performed by the ELISA method

HGrC1 cells were seeded in 96-well culture plates at a density of 5 × 10^3^ cells/well, COV434 cells at a density of 8 × 10^3^ cells/well and KGN cells at a density of 1.2 × 10^4^ cells/well. Leptin receptor antagonists were added at concentrations of 10, 100 or 1000 ng/mL, with leptin at a concentration of 40 ng/mL. Cells were cultured for 48 h with repeated exposure. The culture medium was changed daily and fresh compounds were added. Androstenedione (10^−5^ M) was used as a substrate for oestradiol production. After 48 h, the media were collected and stored at −20 °C until analysis. Oestradiol concentrations in the medium were measured by enzyme immunoassay (EIA) using a commercially available ELISA kit (DRG Diagnostic, Germany). All samples were run in duplicate in the same assay. The analytical sensitivity was 10.6 pg/mL. The intra-assay variation was 8.7–9.23%, and the inter-assay variation was 6.87–14.91%. The range of the oestradiol (E2) assay was 10.6–2000 pg/ml.

#### Statistical analysis

Data were expressed as mean ± SEM from the four independent experiments performed in triplicate. Statistical analyses were performed using GraphPad Prism 5. Data were analysed using a one-way analysis of variance (ANOVA) followed by Tukey’s honestly significant difference (HSD) multiple range test. A value of *P* < 0.05 was considered to be statistically significant.

## Results

### The action of SHLA, Lan1 and Lan2 on leptin-stimulated leptin receptor (ObR) gene and protein in different cell lines

**Fig. 1:**
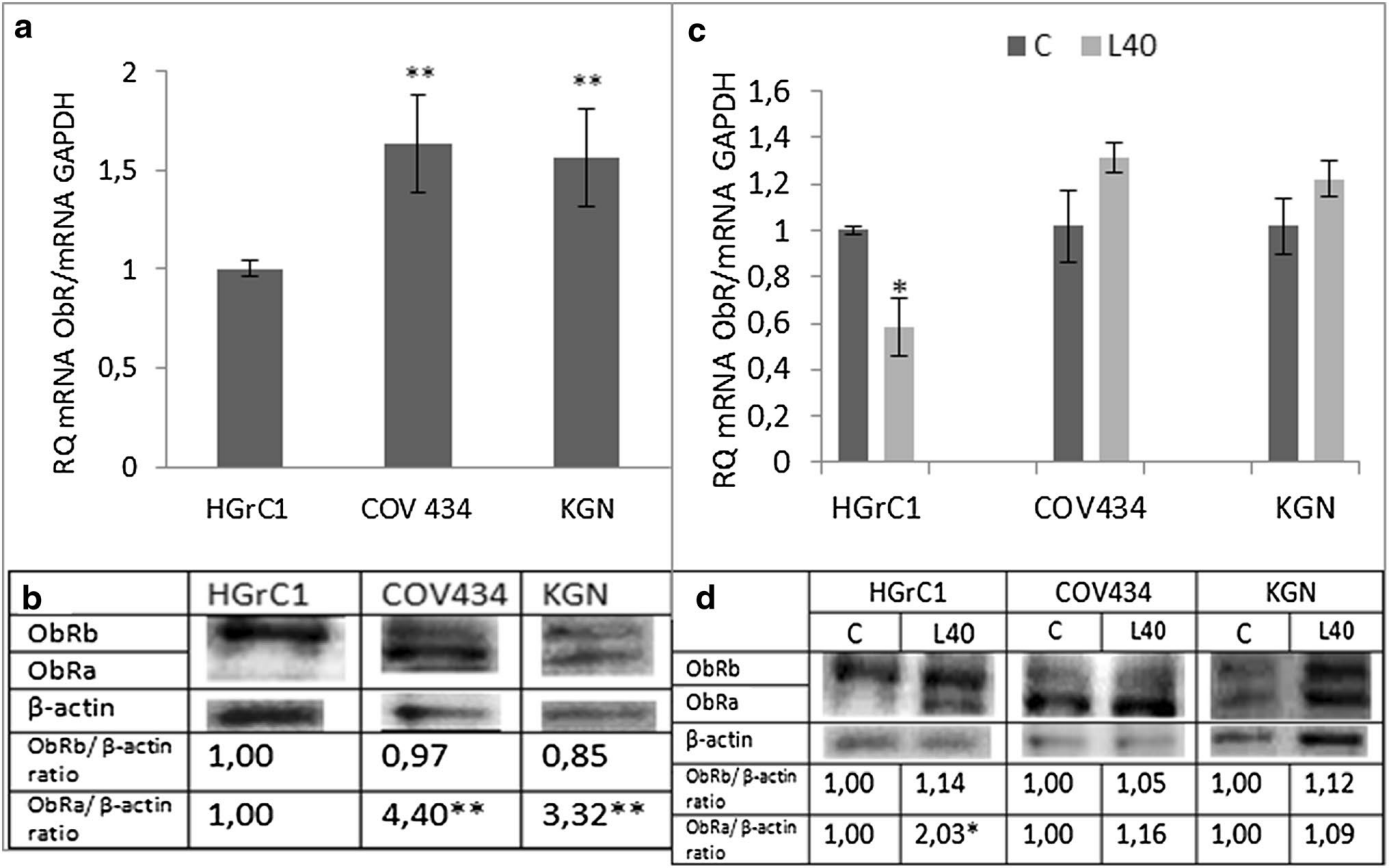
Basal expression of leptin receptor (ObR) **a** gene and **b** protein, and leptin action on ObR **c** gene and **d** protein expression in *different cell lines*. Basal mRNA was evaluated by qPCR after 24 h of cell culture and by Western blot after 48 h. All the results were normalised to HGrC1 (ObR expression) with a value equal to 1. Values are mean ± SEM. Statistically significant differences between groups are indicated by *(*p* < 0.05) and **(*p* < 0.01). Densitometry results were normalised to β-actin loading controls to obtain a band ratio. All values marked with **(*p* < 0.01) are significantly different from the control

The basal leptin receptor gene expression varied between different cell lines. Assuming the gene expression in HGrC1 cell to be 1, ObR gene expression in COV434 and KGN cells was 50% higher (Fig. 1a), while there were no differences in ObR protein expression (Fig. 1b).

In non-cancerous HGrC1 cells, leptin (40 ng/mL) decreased ObR gene expression, had no effect on ObRb, but increased ObRa protein expression. In both cancer cell lines, COV434 and KGN, leptin slightly increased gene expression but had no effect on both ObRb and ObRa protein expression (Fig. 1c, d).

**Fig. 2:**
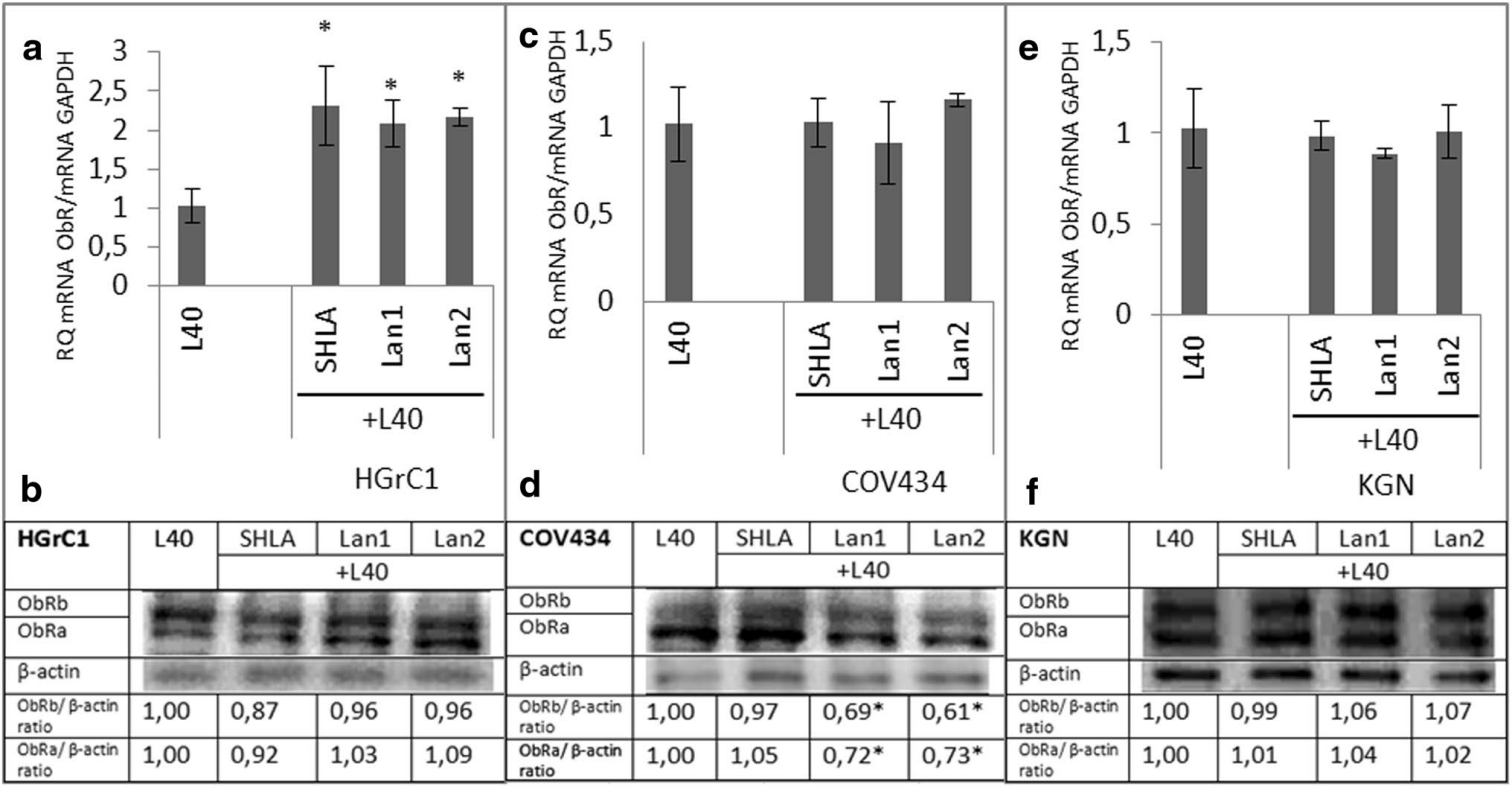
Effect of leptin receptor antagonists (SHLA, Lan1 and Lan2) on leptin receptor (ObR) gene (**a**, **c**, **e**) and protein (**b**, **d**, **f**) expression. All values marked with *(*p* < 0.05) are significantly different from control values. Values are mean ± SEM. Densitometry results were normalised to β-actin loading controls

In HGrC1 cells, all three antagonists increased ObR gene expression (Fig. 2a), reversing the inhibitory effect of leptin on ObR gene expression (Fig. 1c), but had no effect on protein expression (Fig. 2b). None of the antagonists affected ObR gene expression in COV434 and KGN cells (Fig. 2c, e). The inhibitory effects of Lan1 and Lan2 on both forms of ObR protein expression were observed in COV434 cells (Fig. 2d). In KGN cells, none of the ObR antagonists investigated had an effect on ObR protein expression (Fig. 2f).

### The action of SHLA, Lan1 and Lan2 on leptin-stimulated expression of oestrogen receptor (ER) gene and protein in different cell lines

**Fig. 3:**
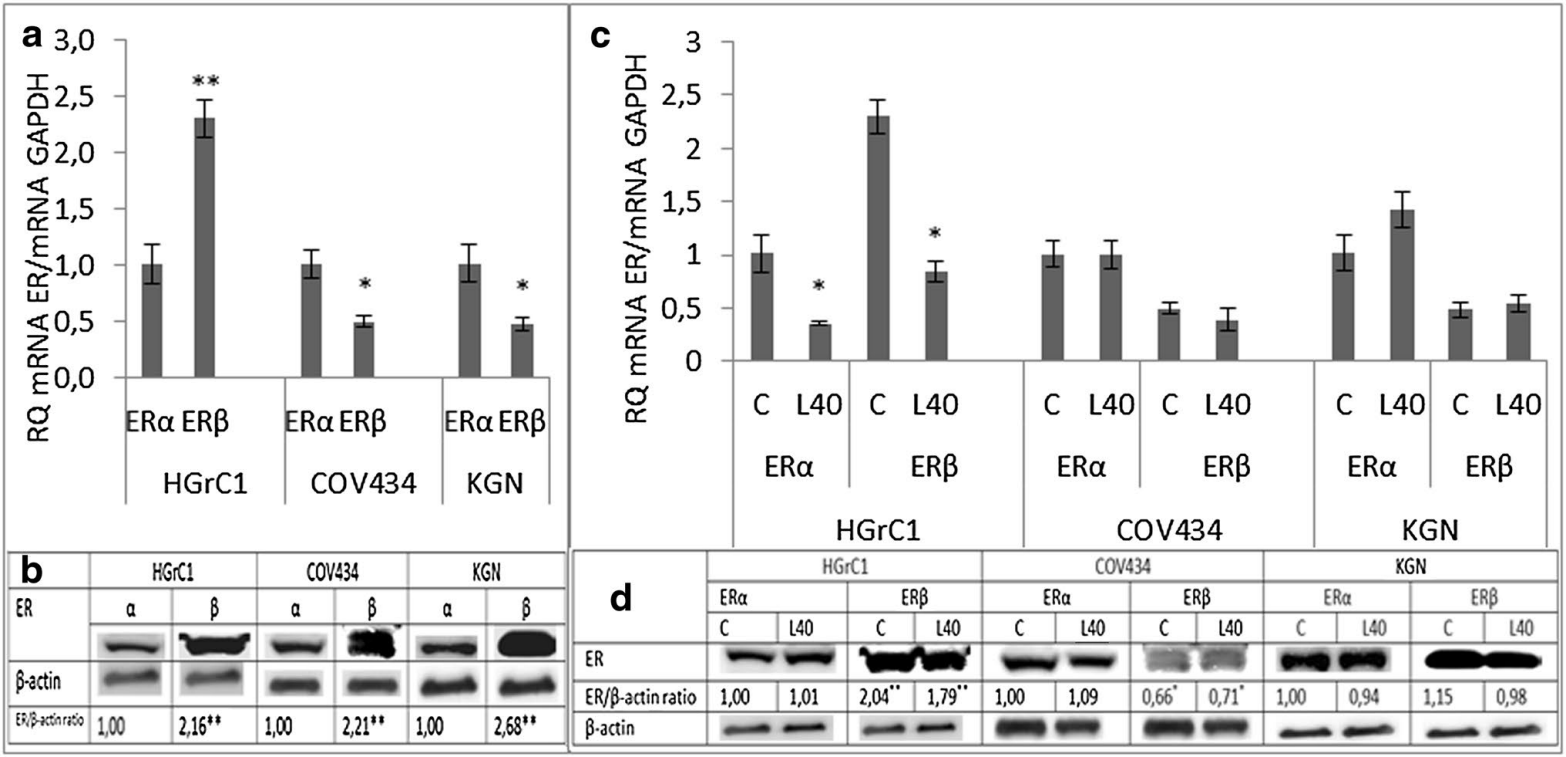
Expression of the oestrogen receptor (ER) α and β forms at **a** the gene and **b** protein levels in *different cell lines* and the action of leptin on ER **c** gene and **d** protein expression. *Each point* represents the mean ± SEM from three independent experiments. All values marked with *(*p* < 0.05) and **(*p* < 0.01) are significantly different between ERα and ERβ (**a**, **b**) or from control values (**c**, **d**). All values marked with *(*p* < 0.05) and ** (p < 0.01) are significantly different from control values. Densitometry results were normalised to β-actin loading controls

The basal expression of the ERβ form in HGrC1 cells was twofold greater than the ERα form at both the gene and protein levels (Fig. 3a, b). In contrast, in the cancer cell lines, both gene and protein ERβ expressions were higher than those of the ERα form (Fig. 3a, b).

Leptin (40 ng/mL) decreased both ERα and ERβ gene expression in HGrC1 cells, but had no effect on protein levels. In cancer cells, the expression of ER gene (Fig. 3c) and protein (Fig. 3d) in COV434 and KGN cells was unchanged by the addition of leptin.

**Fig. 4:**
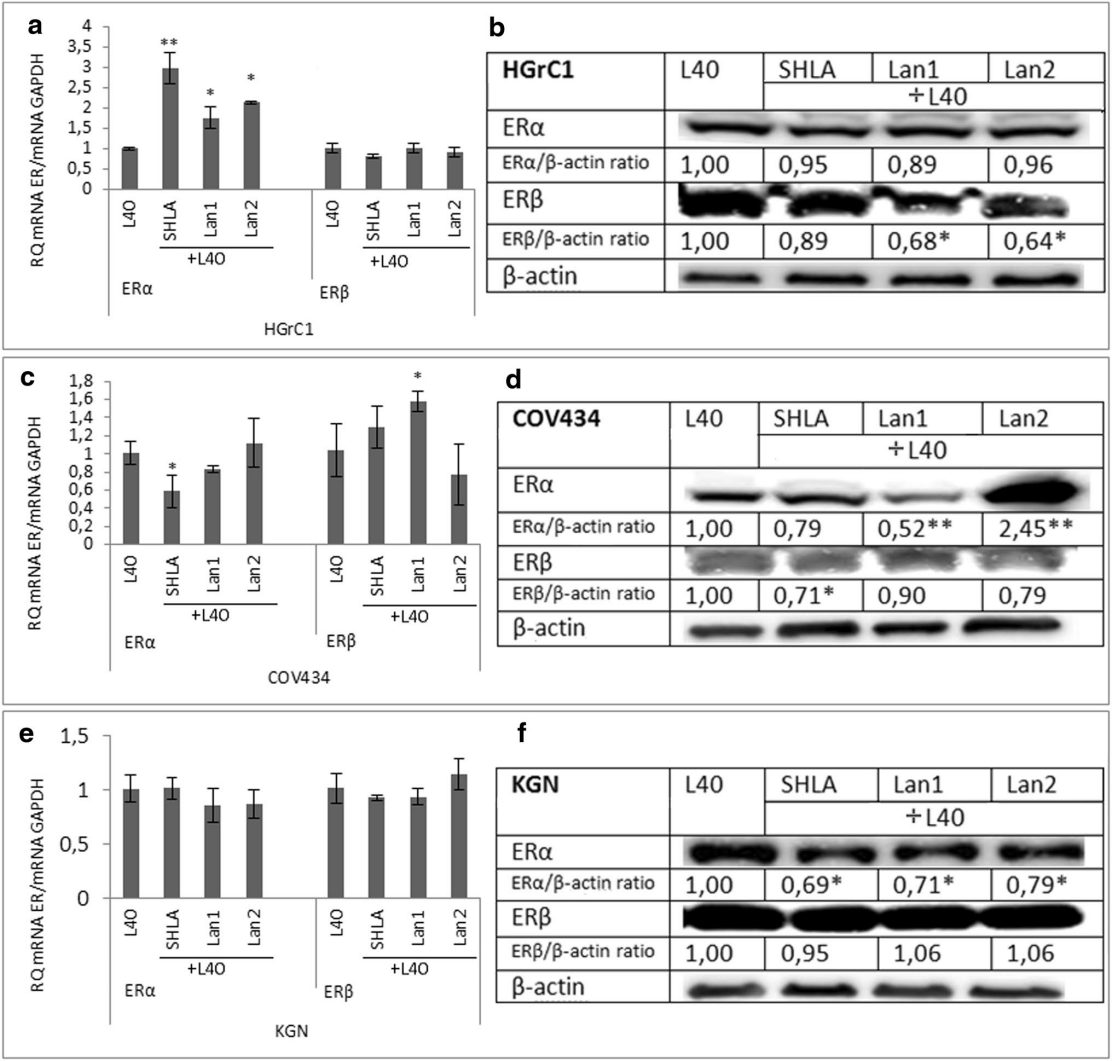
Effect of leptin receptor antagonists (SHLA, Lan1 and Lan2) on the expression of the oestradiol receptor α and β forms on the gene (**a**, **c**, **e**) and protein (**b**, **d**, **f**) level. Values are mean ± SEM. All values marked with *(*p* < 0.05) and **(*p* < 0.01) are significantly different from control values

In HGrC1 cells, all three leptin receptor blockers increased ERα gene expression, but had no effect on ERβ gene expression (Fig. 4a). None of the antagonists affected ERα protein expression, although an inhibitory effect of Lan1 and Lan2 on ERβ protein expression was observed (Fig. 4b). In COV434 cells, SHLA decreased ERα gene expression and ERβ protein expression, but had no effect on ERβ gene expression. Lan1 increased ERβ gene expression and decreased ERβ protein expression, while Lan2 had the opposite effect (Fig. 4c, d). In KGN cells, all three blockers failed to affect ERα gene expression (Fig. 4e) but reduced ERα protein level (Fig. 4f). No effect on ERβ gene or protein expression was observed in KGN cells (Fig. 4e, f).

### Effect of leptin receptor antagonists on proliferation, caspase-3 activity and oestradiol secretion

**Fig. 5:**
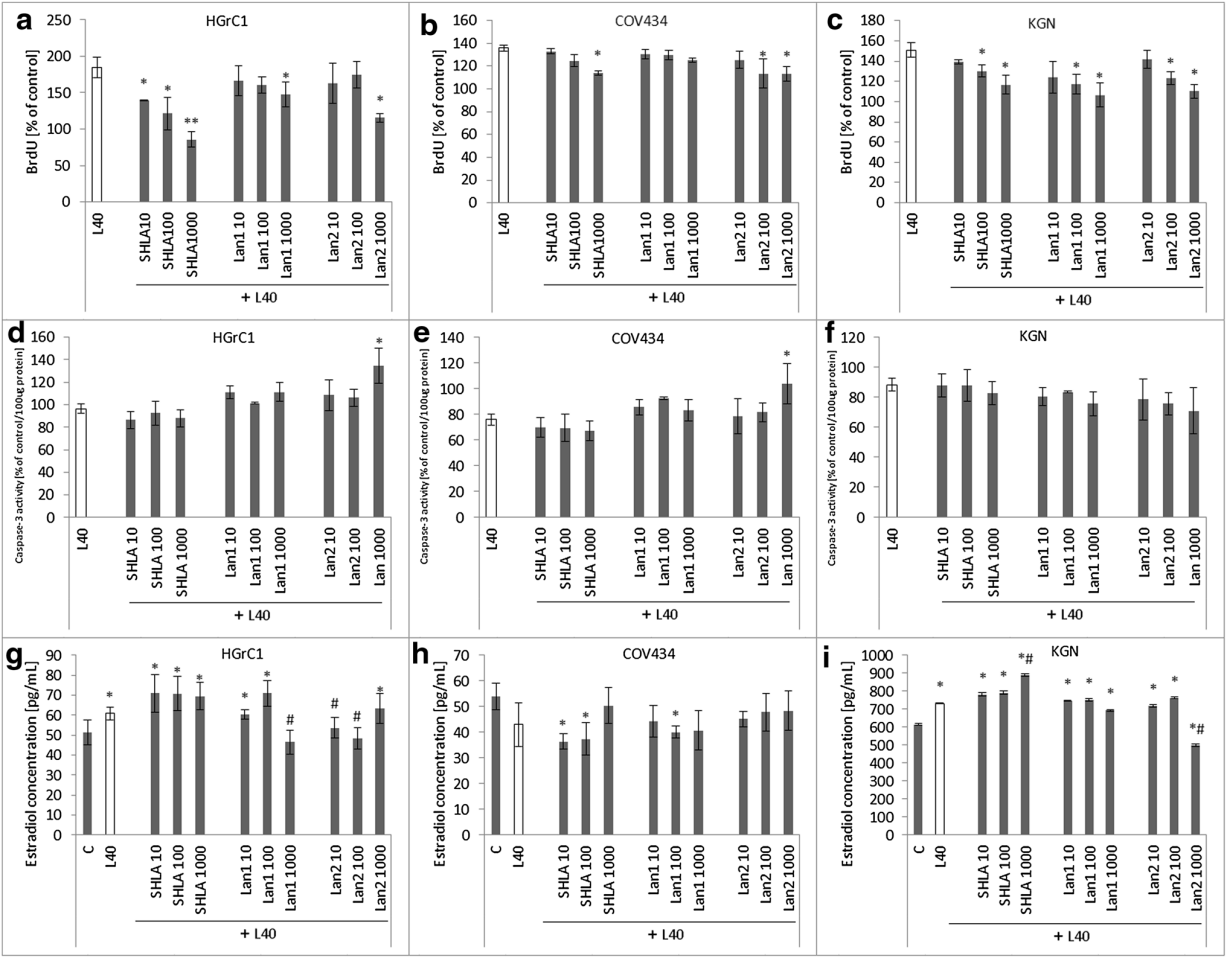
Effect of leptin and leptin receptor antagonists (SHLA, Lan1 and Lan2) on **a**–**c** cell proliferation (BrdU incorporation), **d**–**f** caspase-3 activity and **g**–**i** oestradiol secretion. *Each point* represents the mean ± SEM from three independent experiments of four replicates per treatment group. In **a**–**f,** values marked with *(*p* < 0.05) and **(*p* < 0.01) are significantly different from leptin values. In **g**–**i,** values marked with * are significantly different from control, while those indicated by ^#^(*p* < 0.05) are significantly different from leptin

In HGrC1 cells, leptin increased cell proliferation (80%) but had no effect on caspase-3 activity and slightly increased estradiol secretion (30%). All three leptin receptor blockers decreased BrdU incorporation (Fig. 5a), except that Lan2 at the highest concentration (1000 ng/mL; Fig. 5d) had no effect on caspase-3 activity. Lan1 (1000 ng/mL) and Lan2 (10 and 100 ng/mL) reduced oestradiol secretion (Fig. 5g).

In COV434 cells, SHLA and Lan2 at dose 1000 ng/mL decreased BrdU incorporation, while Lan1 had no effect (Fig. 5b). None of the blockers investigated had an effect on caspase-3 activity (Fig. 5e) or oestradiol secretion (Fig. 5h).

In KGN cells, SHLA at the highest dose and Lan1 and Lan2 at doses 100 and 1000 ng/mL decreased BrdU incorporation (Fig. 5c). None of the three antagonists investigated affected caspase-3 activity (Fig. 5f), except for Lan2, which at the highest concentration decreased oestradiol levels. SHLA and Lan1 had no effect on oestradiol secretion (Fig. 5i).

### Effect on leptin receptor antagonists on selected cell cycle protein expression

**Fig. 6:**
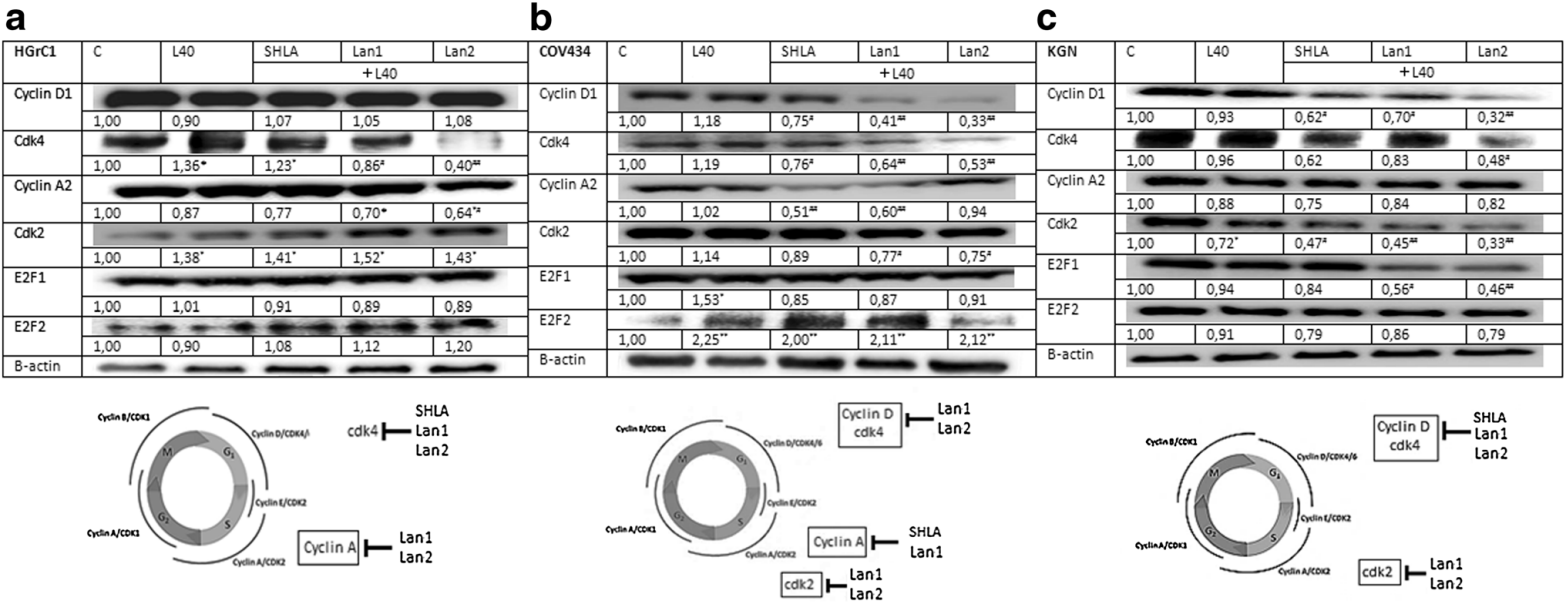
Expression of cell cycle proteins in HGrC1, COV434 and KGN cell lines under the influence of leptin and leptin receptor antagonists. The representative blots of three experiments are shown in the panels. Cdk2, cyclin A, cdk4, cyclin D and E2F1 densitometry results were normalised to GAPDH loading controls to obtain band ratios. Values are mean ± SEM. All values marked with *(*p* < 0.05), **(*p* < 0.01) are significantly different from untreated control values. All values marked with ^#^(*p* < 0.05), ^##^(*p* < 0.01) are significantly different from the values of leptin at 40 ng/mL

In HGrC1 cells, leptin increased cdk4 and cdk2 protein expression and had no effect on cyclin D1 and A2. No effect on E2F1 or E2 was observed. Of the investigated leptin receptor blockers, SHLA had no effect on selected cell cycle gene or protein expression. An inhibitory effect on cdk4 but not cyclin D was noted under the influence of Lan1. A marked inhibitory effect on cyclin A and cdk4 protein expression under the influence of Lan2 was noted. None of the antagonists investigated had an effect on E2F1 or E2 (Fig. 6a).

In COV434 cells, leptin with the exception of a stimulatory effect on E2F2 had no effect on the expression of any other investigated cell cycle proteins. All three leptin receptor blockers decreased cyclin D1 and cdk4 in the following order: SHLA < Lan1 < Lan2. In addition, SHLA decreased cyclin A2 protein expression, Lan1 cyclin A2 and cdk2 expression, and Lan2 cdk2 protein expression. Of the three blockers studied here, only Lan2 had an inhibitory effect on E2F2 expression (Fig. 6b).

In KGN cells, leptin, with the exception of a slight inhibitory effect on cdk2, had no effect on the expression any of the cell cycle proteins studied. All three blockers decreased the expression of cyclin D1 and cdk2, while Lan2 also reduced cdk4 expression and both Lan1 and Lan2 reduced E2F1 protein.

## Discussion

This study clearly demonstrates twofold higher leptin receptor gene and protein expression in cancer granulosa cell lines compared to non-cancer cell lines. Additionally, we found that leptin increased its own receptor gene expression only in cancer cell lines.

These results on both the short (ObRa) and long (ObRb) forms of the leptin receptor are in agreement with other studies demonstrating both forms of the leptin receptor in granulosa cells [11, 24]. As described by us, higher ObR expression and a leptin-stimulatory effect on its own receptor in cancer granulosa cells correspond with our previously published data [14] in epithelial ovarian cancer cells, suggesting similar effects in both epithelial ovarian cancer and folliculoma. Increased expression of ObR, corresponding to higher risk, has also been described in cases of breast [1] and prostate cancer [25], both hormone-dependent cancer types. In ovarian cancer patients, high leptin levels are associated with poor treatment prognosis [26].

Cross talk between leptin and oestrogen receptors should also be taken into consideration. It was apparent that, in cancer cells, elevated ObR expression correlated with elevated ERα expression. The direct relationship between ObR and ER is still unresolved; however, the existence of cross talk between these receptors was examined. Fusco et al. [19] described a threefold increase in ERα expression in the presence of 100 ng/mL leptin. Different effects, dependent on the type of ER expression, have been described by Ray et al. [27] and Ozbay et al. [28], who showed that leptin increased the proliferation level to a greater extent in the ER+ breast cancer cell line T47-D than in ER− MDA-MB231 cells. This finding is in line with our results, as all investigated granulosa cell lines demonstrated a higher degree of proliferation than in previously examined epithelial ovarian cell lines [14]. Physiologically, the primary sites of oestrogen receptors include the same areas where ObR are located [29]. Leptin can enhance aromatase activity, promoting oestrogen production from androstenedione in adipose tissue and hence stimulate the progression of oestrogen-dependent breast cancer [30]. Furthermore, leptin enhances the activation of oestrogen receptor alpha (ERα) through the MAPK pathway in MCF-7 and HeLa cells [31]. In ER+ MCF-7 cells, chronic exposure to leptin has been found to result in a higher ERα/ERβ ratio, enhanced oestrogen transcriptional activity, greater cell growth and resistance to the anti-oestrogen compound tamoxifen [32].

It is well known that leptin can directly affect ovarian function by its action on oestradiol secretion [33]. There have been a few studies on blocking leptin activity in the ovary and interactions with oestradiol secretion. Our results show that the leptin receptor antagonists SHLA and Lan1, at concentrations of 100 ng/mL, increased oestradiol (E2) secretion by the HGrC1 cell line, but had no effect on oestradiol secretion in granulosa cancer cells (except for Lan2, which decreased E2 secretion in KGN cells). The effect on ERα and ERβ expression was variable in this study. In HGrC1 cells, all antagonists decreased ERβ gene expression, but only Lan1 and Lan2 decreased protein levels. The blockers SHLA and Lan1 decreased ERα in juvenile COV434 cells, while Lan1 and Lan2 had an effect in adult form KGN cells. These results suggest that, independently of the magnitude of ERα expression, Lan1 was sufficient in both types of cells. Our data are in agreement with Fusco et al. [19], who showed that leptin receptor silencing in MCF-7 breast cancer cells results in decreased ERα expression. The same authors reported that higher leptin levels are more strongly correlated with ER+ breast cancer rather than ER−. In another study, Dupuis et al. [11] used the leptin receptor antagonist PEG-SMLA to demonstrate that inhibition of ObR impaired follicle rupture without affecting meiotic maturation of oocytes in ovarian follicles.

Leptin, via direct action through ObR and additionally by enhancing the activation of oestrogen receptor alpha (ERα) through the MAPK pathway, induces cell proliferation. The data presented here show that leptin at a supraphysiological level induced cell proliferation in all investigated cell types, with the greatest effect observed in the normal granulosa cell line HGrC1 where proliferation reached 180% of the control. The mutagenic effect of leptin has been observed in various cell types, including breast cancer [33, 34], endometrial cancer [35] and prostate cancer cells [25]. Kato et al. [36] showed that higher leptin levels (above 100 ng/mL) or longer incubation times are required to have an effect on proliferation. However, Fiedor and Gregoraszczuk [14] and Ptak et al. [37], using the same leptin concentration, described a similar effect on the proliferation of epithelial ovarian cancer cells.

All ObR antagonists used in this study reversed the leptin-stimulatory effects on cell proliferation, although with varying degrees of success. In non-cancerous HGrC1 cells, the most potent antagonist was SHLA. In KGN cells, all three antagonists at all concentrations reversed leptin-stimulated proliferation. Surprisingly, in the COV434 cell line, the effects of blockers were negligible. The use of leptin receptor antagonists has been well studied in breast cancer. There are reports of the inhibitory action of Aca-1 and Allo-aca on leptin-stimulated proliferation of MCF-7 and MDA-MB231 breast cancer cells [15], and D-Ser and DDD on the proliferation of MCF-7 cells [38]. Catalano et al. [16], using LDFI (leptin binding site I), showed the inhibitory effects on the leptin-induced growth of ERα-positive (MCF-7) and ERα-negative (SKBR3) breast cancer cells. Fusco et al. [19] demonstrated the inhibitory effects of a neutralising monoclonal antibody (9F8) on cell proliferation in the ER-positive MCF-7 cell line, but not in MDA-MB231 ER-negative cells. We previously described SHLA and Lan2 as a promising treatment for epithelial ovarian cell tumours [14]. To our knowledge, these are the first findings to indicate the possible use of ObR blockers in the treatment of folliculoma cancer.

Our results reveal that leptin at 40 ng/mL does not affect caspase-3 activity in non-luteinising cells HGrC1, but could decrease it in COV434 cells and to a small extent in KGN cells. Using granulosa cells, Sirotkin et al. [39] showed a stimulatory effect of leptin at a dose of 100 ng/mL on Bax protein in human granulosa cells, suggesting that leptin can modulate apoptosis in human ovaries. The discrepancy in the findings may be due to the different concentrations of leptin. All the antagonists investigated here had no effect on caspase-3 activity in HGrC1 or KGN cells. Only Lan1 and Lan2, at the highest concentrations, restored caspase-3 activity close to the control level in COV434 cells. Previous experiments conducted in our laboratory have shown that, in epithelial ovarian tumours, leptin at 40 ng/mL alone or in combination of SHLA or Lan2 does not affect the activity of caspase-3, -8 or -9 (unpublished data).

With regard to the mechanism of action of the ObR antagonists, we showed that, in non-cancer HGrC1 cells, Lan1 and Lan2 decreased the expression of cyclin A2 and cdk4. In COV434 cells, all tested antagonists decreased cyclin D1 and cdk4 protein expression, while Lan1 also decreased cyclin A2 and cdk2, SHLA had an inhibitory effect on cyclin A, and Lan2 inhibited cdk2 expression. It is generally believed that the critical function of the cyclin A–Cdk2 complex is the phosphorylation of substrates that start DNA replication and co-ordinate the end of S-phase [40]. In KGN cells, only cyclin D1 and cdk2 were decreased by all leptin receptor blockers. Additionally, we observed an inhibitory effect of Lan1 and Lan2 on the expression of the transcription factor E2F1, suggesting that the anti-proliferative effect of this antagonist in ovarian cancer may be mediated, in part, by the down-regulation of E2F1. Our previously published results [14] concerning the action of leptin receptor antagonists on epithelial ovarian cancer cells showed that both antagonists studied decreased cdk2 and cdk4 protein expression in CaOV-3 and OVCAR-3 cells. Additionally, in CaOV-3 cells, cyclin D1 expression decreased under the influence of SHLA and Lan2.

In summary, (1) in juvenile form of folliculoma, SHLA and Lan-1 increased ERβ expression, while in the adult form all blockers decreased ERα expression. As a consequence, the ratio moved towards greater expression of ERβ, characteristic of non-cancer granulosa cells. (2) In both types of folliculoma, Lan1 and Lan2 acted as inhibitors of cyclinD/cdk4, cdk2 and E2F. The ability of these cyclins to activate the cyclin-dependent kinase CDK4 is the most extensively documented mechanism for their oncogenic actions and provides an attractive therapeutic target.

In conclusion, taking into consideration that these results are based on experiments performed on cell lines, and did not consider the tumour microenvironment, further studies should be performed using explants of granulosa cancer from patients with folliculoma or the coculture of granulosa cancer cell line with fibroblasts, epithelial cells and other components of the tumour environment.
